# Diagnosing dementia: Ethnography, interactional ethics and everyday moral reasoning

**DOI:** 10.1057/s41285-016-0018-x

**Published:** 2016-09-21

**Authors:** Alexandra Hillman

**Affiliations:** School of Social Sciences, WISERD, Cardiff University, 45 Park Place, Cardiff CF10 3BB, UK

**Keywords:** UK, dementia diagnosis, sociology of medical ethics, ethnography

## Abstract

This article highlights the contribution of ethnography and qualitative sociology to the ethical challenges that frame the diagnosis of dementia. To illustrate this contribution, the paper draws on an ethnographic study of UK memory clinics carried out between 2012 and 2014. The ethnographic data, set alongside other studies and sociological theory, contest the promotion of a traditional view of autonomy; the limiting of the point of ethical interest to a distinct moment of diagnosis disclosure; and the failure to recognise risk and uncertainty in the building of clinical ‘facts’ and their communication. In addressing these specific concerns, this article contributes to the wider debate over the relationship between sociology and bioethics (medical ethics). At the heart of these debates lies more fundamental questions: how can we best understand and shape moral decision-making and ethics that guide behaviour in medical practice, and what should be the guiding ideas, concepts and methods to inform ethics in the clinic? Using the case of dementia diagnosis, this article illustrates the benefits of an ethnographic approach, not just for understanding this ethical problem but also for exploring if and how a more empirically informed ethics can help shape healthcare practices for the better.

## Introduction

Given the centrality of autonomy, beneficence and informed consent with regard to issues of diagnosis disclosure to modern bioethical thinking, it is unsurprising that scholars in this area have found diagnosis disclosure in dementia particularly challenging. For example, scholars have tended to focus on issues that are high on the public agenda or are particularly extreme (Musschenga, 2005), with much of the ethical debate centred on advanced directives or debates over the right to die (DeGrazia, 1999). In a similar vein, issues relating to dementia diagnosis have tended to focus, not on current practice, but on a potential future in which neurodegenerative diseases, such as Alzheimer’s disease, can be diagnosed with a high degree of accuracy, pre-symptomatically, allowing for the extremes of the debate to come to the fore (see, for example, Powell, 2014). Focussing on the extremes of the argument is symptomatic of the epistemological grounding of ethics in moral philosophy, which relies on systems of argumentation to develop its theory. This is a practice better served using the tools of imagined or hypothetical scenarios that can be driven by theory rather than the detail and nuance of an actual clinical case. Principles, therefore, take priority in the sense that they justify or criticise practice (Arras, 1991).

The relative lack of attention from ethicists to the issue of current diagnoses disclosures in dementia is based on two key assumptions: firstly, that clinicians have an ethical imperative to inform patients of their diagnosis, unless they choose not to know (Marzanski, 2000) [at least this is the case in western medical practice (Henrique, 2003)] and, secondly, that the evaluation of practices of disclosure (that is the way in which a diagnosis is given) is the subject of psychologists or other disciplines that focus on the analysis of communication and its effects. Subsequently, the question of *whether* to tell people their diagnosis is perceived to be a question of ethics but the question of *how* such a diagnosis is shared is assumed to be a question for social science.

The power of concrete principles through which to develop guidelines for medical research and practice has arguably intensified over the past thirty to 40 years in the UK, resulting in what Reubi (2013) has described as an increasingly influential ‘bioethical thought collective’. This article illustrates how ethnography can inform the framing of an ethical debate in relation to the diagnosis of dementia, developed not through principles, but through the study of practices. By doing this, the paper contributes to a wider body of work that highlights the contribution of empirical social research and sociological theory, to the understanding of moral and ethical practice in medical work (Haimes, 2002; Hedgecoe, 2004). The view of the individual as the truest measure of ethics that transcends culture has been historically challenged by sociologists who recognise society, social structures and cultures as both informing and, more significantly, producing moral norms. As a consequence, sociologists have looked to social practices through which moral norms are produced to challenge, shape and inform ethics. Such an approach privileges practice and experience and challenges a bioethics that seeks to develop moral codes based on the philosophy of individual thought and action. Taking this as its starting point, this paper builds on the contributions of Cicourel (2006, 2011, 2013), Beard (2008), Beard and Neary (2013), Beard and Fox (2008) and Moreira (2010) in particular, who have undertaken ethnographic work in memory clinics, to better understand ethics and moral understandings in the context of dementia diagnosis.

## The Ethnography

This article draws on an ethnography undertaken in two memory clinics in the UK, carried out between 2012 and 2014. Both clinics were based in large university teaching hospitals, one in a city location, the other located in a rural area. Both memory clinics functioned in very similar ways, assessing patients experiencing problems with thinking and memory. The most common route through which patients attended the memory clinic was through referral from their general practitioner (GPs). Other routes included referral from another community or primary care service such as day centres, or less commonly from secondary services or by referral by patients themselves or by their relative or carer. Following referral, patients have an initial assessment. This assessment involves the following: cognitive tests (most commonly undertaken in a separate adjoining room by a psychologist or nurse practitioner but sometimes carried out by the doctor or psychiatrist as part of the consultation); the taking of a detailed patient history by asking questions of the patient themselves and their relative/carer; and clinical tests – some done on site that day, others arranged for a later date – including blood tests (mostly done to exclude any other potential clinical cause of their memory problem), a trace of the heart if it is a possibility that the patient may require medication for their memory which carries contraindications for some heart arrhythmias and, increasingly, a Computerised Tomography (CT) scan of the brain.

The fieldwork was made up of observations in the memory clinics. This involved both the audio recording of clinical consultations, alongside observations and the taking of fieldnotes of the encounters. This approach captured talk and interactions involved in initial patient assessments, the discussion of test results and processes of diagnoses as well as the broader social, material and spatial contexts in which these encounters took place. Over the periods of observation, 51 consultations were observed. As well as the in-clinic observation, the researcher interviewed 13 memory clinic staff, 21 patients who had attended a memory clinic, 19 relatives/carers (10 of the patients and relatives were interviewed twice and one couple were interviewed three times) and 10 research experts working in the field of dementia. Due to the focus of this article, the material presented focusses on the in-clinic consultations and the accounts of memory clinic staff.

Analysis, as with most ethnography, began in the field, interpreting the social meaning of actions and interactions and situating them in their wider contexts. The fieldwork process intended to make visible the practices of valuing itself, to show the production of morals in the everyday actions and interactions that occurred in the clinic. All ethnographic research, to some extent, implicitly impinges on ethics, as its practice implies the evaluation of persons, events, motives and consequences. The approach of this ethnography was to reject the mystery of the moral order within (as Kant so famously described it), and instead to focus attention on the ‘moral order without’ (Garfinkel, 1964), to pay attention to the collaborative production of moral orders and to show how moral understandings shift together, in many daily interactions of social life (Walker, 2010). Paying attention to the production of morals in clinic interactions or in clinician’s accounts of their practice is therefore essential to understand what specific kinds of ethics are produced in memory clinics and in what circumstances.

Selected examples from the field and from interviews have been chosen on the basis of their capacity to exemplify the concepts, justifications and explanations of clinicians and/or patients and families, which enable them to make sense of the social situation. They have also been chosen to help illustrate how social practices and their material form help accomplish what is ethical in the context of dementia diagnosis. In making these selections, the researcher is of course engaged in a constant dialogue between the interpretations of actor’s sense-making practices and their own interpretation of social theory and other social studies of dementia or memory clinics. Such a dialogue is further mediated by the researcher’s own participation in the same conversations and encounters and bringing to them their own set of experiences. In a sense, this is the very essence of ethnography, to utilise the mundane practices of sense-making through which we, as social actors, experience and participate in the world as a tool for interpretative analysis (Ingold, 2014).

## Dementia Diagnosis and Interactional Ethics

The shared contribution of previous ethnographic work in memory clinics (Cicourel, 2006, 2011, 2013; Beard, 2008; Beard and Fox, 2008; Beard and Neary, 2013; Moreira, 2010), which of course have differing concerns and interests, is to show how there are tensions in memory clinics regarding the location of memory, which can be condensed into an individual’s body – their brain – while simultaneously distributing memory loss to the people and communities surrounding the person (Moreira, 2010). Highlighting this tension is significant for understanding the moral and ethical framing of the diagnostic process in the memory clinic, to show the multiple agendas being enacted and the necessarily collaborative processes through which a decision regarding the causes of memory loss are reached and communicated. Building upon these previous contributions, this work challenges the restriction of ethical interest to a single point of diagnosis disclosure, highlighting the social, collaborative and processual nature with which a diagnosis is reached and the production of ethics that emerge through the course of these social practices.

### The signicance of the patient and relative’s story

A significant contribution of ethnographic insight into the practices of dementia diagnosis is to highlight the experiences of those attending memory clinics and to forefront patients’ stories of memory loss or cognitive decline. As Beard and Fox’s work (2008) shows, processes of diagnosis require a transition to occur from everyday forgetfulness – the patient’s experience – to a medical problem – a symptom. Attending to this process is important in ascertaining the meanings attached to memory by patients themselves but also in recognising the ethics that are imbued in the transformation that occurs from the patient’s story, to clinical symptoms and finally to a medical explanation. In this study, patients’ perceptions regarding their capacities to think and remember are shown to be shaped by their being in the world: their physical, social and cultural environment, as the following extract exemplifies:

The doctor tells me that the next appointment is with a man in his seventies who has a history of depression but has been referred to the clinic by his GP due to experiencing difficulties with concentration and memory. The man knocks on the door of the assessment room, enters slowly and sits on the chair opposite the doctor. Following brief introductions, the doctor asks for his perspective of why he’s there,

Doctor Talk me through it from your point of view.

Patient Well, I was – I retired early.

Doctor Yeah, when was that?.

Patient Oh, when I was 60. I’ve been travelling round the world ever since, in a motor home.

Doctor Fine, sounds good to me.

Patient The last couple of years, because I lost confidence in myself and I have a wife, and I didn’t want to cause any problems ‘cause the roads get faster, everything gets faster and I was slowing down. So I decided it was time to knock it on the head and – so we live in a caravan now.

The conversation continues with little in the way of direct explanations offered by the doctor and ends with a plan of further tests and the suggestion of a change of medication for his mood.

In this extract, the patient’s description of his problem is framed by the social and temporal characteristics of his environment – ‘everything gets faster’ while he was slowing down. His description of experiencing difficulties with his thinking is manifest in his interactions with the social world in which he is embedded. The account of this man is indicative of the distinction made by Beard and Fox (2008) between the patients’ experience, that is likely to be socially shaped, and the transformation that is required for this to become symptomatic of a medical problem. Similarly, memory – or lapses in memory – often require social interaction with others to identify them as something out of the ordinary, as a problem to be addressed:

Later that afternoon we see a lady in her early 80s. Although she is quiet and pleasant, she makes it quite clear that she sees little value in being at the clinic or in the assessment process. She sits with her arms crossed, opposite the doctor, with her husband sat on a chair next to her. The doctor begins by asking about what brought them to the clinic,

Doctor Perhaps what instigated you to go to the memory team and what the concerns were?

Patient Right. Well all I can say is my husband advised it and I was a bit surprised because I always thought I had a pretty good memory, but when two or three times it happened that he said, “Oh remember so and so?” and I said, “I can’t remember.” You know, that’s the only reason actually my husband was interested to find out why, why it was like it.

Doctor So did you go to the GP initially?

Patient No, I hadn’t done. It’s just my husband just decided it.

Doctor So you contacted the memory team directly?

Patient Right. I presume my husband did [laughs].

Doctor So it’s been more would you say other people perhaps that have –

Patient I think so.

As the consultation progresses, the doctor hints at possible explanations, suggesting that this might be more than the consequence of getting older and that they have to consider whether this might be a form of dementia. They agree, although the patient herself is somewhat reluctant, that she will have a CT scan and return to the clinic following the scan when they will discuss the issue further.

This extract reflects the social nature of the dementia experience, highlighting the breaking of social norms and expectations that are often only recognised by the patient themselves through their interactions with the people around them. Similarly the extract below is a reflection of many examples of interactions in the memory clinic in which the patient’s account of their ‘problem’ is inextricably bound up in their relationship with important people in their lives:

An elderly couple have come to the memory clinic for a second time for the purposes of checking whether the problems they have been experiencing have deteriorated. The husband, who has memory problems, is smiley and reflects positively about his life and situation. His wife sits and listens to his account but shows more signs of concern, wringing her hands together while her husband speaks.

Doctor How have things been since you last came up in May?

Patient No problems as far as I’m concerned.

Doctor No, okay. Are you noticing any problems with the memory at all?

Patient I do forget things. My wife, she remembers everything.

The patient looks over to his wife who is sat next to him and offers a smile, she smiles back although doesn’t manage to erase the worried expression that had preceded it. The consultation ends with the couple being told that it could now be confident that this is probably Alzheimer’s disease, given the progressive nature of the problems the patient is experiencing.

Close family relationships, and marital relationships in particular, shape the content of patient stories in the clinic so that a memory ‘problem’ is determined to a large extent by the nature of these relationships and their everyday functioning. This patient’s wife remembers for them both so that his forgetting ceases to be a concern for the patient himself. This also has ramifications for a person’s experience of the condition as the effects of dementia’s symptoms on the person with the disease have been shown to be influenced by the perceptions and responses of people around them (Langdon *et al,* 2007).

### Constituting personhood and relational autonomy

Recognising that patients’ experiences of memory are situated, embodied and shaped by their interdependent relationships with others is significant because it challenges some assumptions bound up in the traditional meaning of autonomy, which remains a dominant ethical discourse within bioethics, medical practice and within media representations of medical ethics (Hedgecoe, 2004). Furthermore, respect for autonomy, as a principle of bioethics (see Beauchamp and Childress, 2013), is central to the ethical framing of diagnostic disclosure in dementia (Pinner, 2000). There is however widespread criticism of mainstream bioethics, from within its own discipline and from those observing it, for overemphasising the significance of autonomy, for its asocial framing of individual agency and for being increasingly aligned to an Anglo-American cultural preoccupation with individualism (Dekkers, 2001; Holm, 1995; Fox and Swazey, 2008; Zussman, 1997; Christman, 2004; Code, 1991). Observational engagement, of the kind utilised by ethnographers, can help to re-examine representations of personal autonomy by recognising its capacity, in its universalist form, to (re)produce ideas of personhood that shape everyday practices both within and beyond the clinic. The dominance of this specific conceptualisation of autonomy can get reproduced in the accounts and practices of those working in the clinical domain, as this extract from an interview with one of the doctors in the memory clinic team illustrates:

Ethically this is his information he is the one with the disease. I say that said, you know if it becomes apparent that it’s very distressing to him them I’m not going to persist and go on about it but equally he has a right to know.

Here, the staff member describes the rights of the individual – as an autonomous agent – who should be fully informed of their diagnosis. Of course, this is muddied by the possibility that this knowledge could cause distress, as well as the difficulty of knowing what a diagnosis actually means for the patient and their future, thus calling into question the very possibility of being ‘fully informed’ (Corrigan, 2003). Nevertheless, the staff’s account of their practice remains framed by the view of personhood constituted through this particular conception of autonomy.

Dementia itself is a condition that challenges a view of the person as being defined by particular sorts of mental activity (Hughes, 2001). Agency (the capacity to determine and act upon one’s wishes) is claimed as an essential component of individual autonomy in its traditionalist form (Beauchamp and Childress, 2013), reflecting the philosophical traditions of Kant, Locke and Hume in which the person is constituted as an intelligent being that has reason and reflection. Personhood in this regard is made up of particular psychological states and their continuity and connection. For those with dementia, whose illness can disrupt psychological continuity and connection, between past memories and present events for example, this meaning can result in a loss of personhood (Hughes, 2001).

This meaning of autonomy – that constitutes personhood in this way – is particularly problematic for valuing the lived experience of those with dementia and subsequently for shaping an ethical practice for dementia diagnosis that ensures the personhood and worth of those with dementia is maintained. Jenkins (2014), for example, argues that the conceptualisation of personhood as a bounded, distinct and unique entity is so entrenched in western contemporary culture that the preoccupation with an individualised self has been replicated in the development of ‘person-centred’ dementia care, which aims to ensure the person with dementia is treated as an individual:

I suppose the thing is that there is no text book rule, you just manage every person as an individual really.

Although that person isn’t me, that person isn’t my Mum or Dad, they’re different people, and they’re individuals.

In these two extracts, taken from interviews with memory clinic staff, recognition of the individual is highlighted as being central to the ways in which staff account for the ethics of their clinical practice. However, it is less clear what ‘the individual’ in these reflections consists of. What makes up an individual person in the context of ethical practice in communicating a dementia diagnosis? Person-centred care has been an important keystone in the improvement of treatment and care for those with dementia, developed to a large extent in response to Kitwood’s (1997) promotion of personhood. Although recognising these improvements, Jenkins problematises the particular conception of the individual (as fixed and stable) that is often bound up in the discourses and practices of person-centred care, that fail to recognise personhood as a state of becoming, one that is produced through interactional encounters. Interestingly, this concept of the individual was also challenged by Kitwood himself, who recognised personhood as essentially relational, so that social interaction is constitutive of personhood.

In the case of memory clinic staff, there is a continual tension expressed in their accounts of ethical practice. On the one hand, clinicians reflect philosophies of the person that are bound up in the codes of ethics they are most familiar with, which construct patients as asocial individuals, with the capacities to make free and reasoned choices based on full information. On the other hand, clinicians also construct personhood in a way that reflects their cumulative experience of engaging with patients and families experiencing dementia, witnessing first-hand the difficulty of disentangling the patient – and their diagnosis – from the perceptions, needs and concerns of their loved ones. This tension is reflected in the extract below taken from an interview with one of the geriatricians working in the memory clinic:

I mean I haven’t got much problems of discussing thing with the relatives as well. Because the relatives are obviously, they’re directly involved. They need information about the illness and I think they need to be told the diagnosis sort of clearly. And sometimes they are the only ones that understand what you are talking about. So really yes, it has to be patient’s confidentiality and all respect for the patient but in some, I think in some cases you have to be sensible. And I don’t think you act for the best interest of the patient sort of explaining to carers or giving a diagnosis to carers. At the same time sometimes or even on the same sort of clinic sometimes you’re putting the picture, the carers even a bit before the patients. And you know as far as you’re sort of honest with the patient, as much as the patient wants to know.

Responding to these very tensions, one of the most significant challenges to the traditional meaning of autonomy within bioethics has come from feminist ethics, a scholarly tradition that straddles sociology and ethics, and its proposed alternative concept – relational autonomy. Advocates of relational autonomy point to a need to rethink the concept as a characteristic of persons who are ‘emotional, embodied, desiring, creating and feeling as well as rational’ (Mackenzie and Stoljar, 2000). Although a helpful reconceptualisation for troubling notions of what it means to be autonomous, this work is less concerned with the development and application of abstract ethical concepts, but is instead interested in the ways in which moral understandings are produced over time, in the course of clinical encounters.

### Personhood as the production of social practices

This study therefore seeks to embed itself in social practices, to show the ways in which personhood is maintained through social relations. Cicourel’s (2013) study, for example, shows the ways in which caregivers of people with dementia perform socio-cultural ‘scaffolding’, helping their loved ones to maintain competency in social life and to stabilise their social identity. Such scaffolding practices include supplying leading questions that help give an appearance of a speech event. An example of this practice is shown below in a husband and wife’s response to a clinician’s question about coping with memory lapses. The patient originally looks perturbed by the question and there is a momentary pause before the wife takes the initiative to enable him to respond:

Wife You do write things down and refer to things, like your diary, don’t you dear?

Patient Yes that’s right.

Such practices are relied upon by patients to sustain ‘appropriate’ or ‘expected’ social interaction, thus simulating a sense of ‘normal cultural stability’ (Cicourel, 2013). These interactions are evidence of the ways in which autonomy – if understood to be a central component of our sense of self – must be produced and maintained in social practices. This not only points to the necessity to rethink the meaning of autonomy (as scholars have in the promotion of relational autonomy), drawing more on ideas of relatedness, but also highlights the ways in which ethics are themselves produced, maintained and challenged in everyday interactions betweeen social actors. Contrary to an ethics that assumes the dominance of knowledge, derived by theory over practice, ethnographically informed qualitative sociology shows instead how practice is the method through which knowledge, normative order, rationality and meaning are accomplished (Lynch, 2001).

### Dementia diagnosis and everyday moral reasoning

There is a strong tradition of ethnographic work that explores the everyday accomplishments of moral decision-making in the clinic. Hoffmaster’s (1992) case for the benefits of ethnography to the field of medical ethics, for example, expertly presents the small, pragmatic strategies employed by practitioners to navigate moral concerns. More specific to the field of dementia, Beard (2008) describes how moral reasoning, such as decisions regarding how and when to deliver a diagnosis, or if and how to use the Alzheimer’s Disease label, are framed by organisational cultures within memory clinics including the object of trust (invested in either individual clinicians or a collective sense of medical expertise) and the framing of conditions such as Mild Cognitive Impairment (MCI) (as either a chronic condition to be managed or a scientific puzzle to solve). Similarly, in the current study, the disciplinary cultures of the memory clinic staff are shown to shape their approach to assessments, diagnosis and hence the ethical framing of the clinical encounter. The following extract is taken from an interview with a neurologist who describes the difference between how she approaches the diagnostic process, built, as she explains, through her disciplinary training:

R I suspect that there are large swathes of people who are and again there are all these psychiatrists who dispense the pills and say you’ve got brain rot, you’ve got brain rot and everything looks like Alzheimer’s.

I Do you think there’s still quite a lot of misdiagnosis?

R Yeah definitely, definitely. So we used to see lots of, well I think I saw quite a few interesting people come through that clinic and I’m not putting myself up but I think that when you approach it in the light of what is wrong with this person? Rather than is this Alzheimer’s or not? You’re focus shifts…To a certain extent it depends, I suppose my way of practising is very, it’s quite artisan and small scale, finickity. I see a few people and do everything I can for them rather than Henry Ford production line model of everyone’s the same ultimately.

This extract illustrates how disciplinary cultures, as well as organisational cultures, play a significant role in the framing of the consultation and mediates clinicians’ conceptions of and approach to their ethical practice in relation to diagnosis; in this case, the ethical impetus is on the integrity of the diagnosis itself, and the methods through which to achieve it, rather than if, when or how it is delivered. This is illustrative of the implicit (and explicit) moral calculus that informs clinical practitioner’s everyday actions and decision-making (Featherstone *et al*, 2006). Furthermore, this calculus is made up of many competing systems of categorisation that represent clinical, organisational and societal concerns (Bosk, 1979; McHugh, 1970).

This study shows how, in the course of clinicians’ interactions with patients and families in memory clinics, practitioners’ employ similar kinds of pragmatic strategies to those described by Hoffmaster (1992) to navigate ethical concerns regarding the sharing of clinical information. Time is a particularly useful resource for clinicians in the memory clinic. The uncertainty of a dementia diagnosis, the insidiousness of its developing symptoms and the lack of a time-critical cure can create the potential for practitioners to bide their time, to use time as a resource through which to provide a potential practical solution to the ongoing concern regarding the communicating of a diagnosis. The biding of time was therefore often recounted as a means of responding to moral dilemmas, as illustrated in this extract, taken from an interview with a general practitioner half way through a year’s placement in the memory team:

With this particular condition it’s not as if I have a cure to be able to give you now and so I’m stopping things happening from here so we actually have got time on our hands to be able to wait 8 months to give that diagnosis, ‘cause actually there’s nothing much you can change.

This account highlights the resources that time can offer in navigating ethical decisions regarding if, how and when to provide patients and families with information. Of course the ability to bide your time in the context of the memory clinic was described by some as being under increasing threat, as one of the specialist nurses working in the memory clinic described:

The label is put on because you feel under pressure not to give another appointment.

The increasing numbers of people accessing memory clinic services and the growing public awareness with regards to dementia medicines were both described by clinic staff as placing more pressures on them to diagnose promptly, to free up appointments for new patients as well as to give people access to medication. Such pressure potentially undermines the utility of time, as a pragmatic tool through which to navigate ethical issues regarding if, when and how to communicate a diagnosis.

Clinical tests and examinations also offer practitioners tools through which to foreground particular kinds of clinical information, at particular moments. In the memory clinic, the presentation of the CT scan is a good example of how results of clinical tests can be made present or absent at particular moments to aid clinicians in the everyday navigation of ethical concerns. Decisions regarding if, when and how a CT scan is brought into the consultation process provide clinicians with tools to reassure patients and families; provide greater certainty to a difficult diagnosis; or, conversely, to re-emphasise uncertainty and the importance of time and clinical judgement:

As the conversation continues with the couple (see the extract on page 9 which describes an earlier moment in the consultation), the doctor gives the diagnosis of Alzheimer’s disease, pointing to the changes on the CT scan which is left displayed on the wall above the table where the doctor and the couple sit,

Wife Are you saying that it could develop into Alzheimer’s?

Doctor I think it probably has Mrs Jones, is what I’m saying. I think there’s a line you know.

Patient It’s started.

Wife Is there anything that he can take to slow it down?

Doctor Well I think this is why we need to discuss this at this point really. So there are medications for Alzheimer’s. So I think what’s happened here we’ve had progressive memory problems. There comes a point, particularly when you begin to see the changes on the CT scan, that we’ve got enough information to say that this is probably Alzheimer’s Disease.

In this example, the CT scan was brought into the consultation to help secure a diagnosis of Alzheimer’s disease, to signify and visually represent a change from what was previously described as ongoing memory problems to now being described as a degenerative brain disease. Drugs and medication were also described by some memory clinic staff as a resource through which they were able to soften the communication of a diagnosis, shifting the focus away from a diagnostic label and instead to discuss treatment and intervention plans, as described by one of the clinic nurses:

You know, we say that we’re a bit… also not to make a big deal of it, but just to mention, you know, that it could be this and, you know… and we use tablets, I’ve got to say, as a thing to be positive. You know, that we think that it could be an early Alzheimer’s type condition, but we’ve got these tablets that can help.

Finally, the consultation process itself – and the developing interactions that occur between patient, family and clinician – allow practitioners to probe families, gaining insight into their expectations, preparedness or anxieties regarding the potential information or diagnosis that might be shared, as this extract from a memory clinic clinician describes:

You can gauge from the beginning whether they think they’ve come here for a dementia diagnosis or whether they think oh there’s something going on, I’m not quite sure. And you try and work out how much information they want and how much information they already have and how much they’re already looked up. And, you know, have they kind of looked up and they know it’s the diagnosis and they just want you to confirm it, or whether they actually have no idea whatsoever and they’re not quite sure why mum’s a little bit disorientated…if I’m very convinced and it’s very obvious that there is something more significant, then, you know, we say float the idea. So usually again I float it with the carer first depending on how the patient is.

Understanding and assessing a person’s memory problems, reaching a diagnosis, communicating a diagnosis and prognosis and making decisions regarding future treatment and interventions are made in processes of negotiation and collaboration between patients, families and physicians (Hansen *et al,* 2008). Focussing on a distinct moment of diagnosis disclosure therefore fails to recognise the negotiated nature of ethical decision-making that occurs over time and in collaboration (Fox and Swazey, 2008; Zussman, 1997), and the ethical interest in broader contextual issues beyond the ‘moment’ of disclosure.

Sociology of medicine, in particular, illustrates how diagnoses are social practices and, as such, involve a process of interaction between actors – a process that begins with a patient’s story which juxtaposes and merges with the doctor’s story in order for a diagnosis to materialise (Goldstein Yutel, 2011). During this process, significant decisions are made that inform the moral and ethical content of clinic interactions. For example, in the case of dementia diagnosis, decisions regarding what assessment tools to use, and at what stage of the assessment process to use them, are as much dependent upon ethical concerns as they are clinical judgements. These may include concerns regarding the patient and family’s capacity to understand the tool and its purpose, or the need to balance the clinical effectiveness of an assessment tool with the potential harm it may cause. Furthermore, patients and families are more than passive recipients of information; their interactions with physicians inform clinical evaluations and shape the ethical content of clinical interactions in multiple ways (Balint, 1964; Leder, 1990). A patient’s denial of symptoms, a patient and/or family’s desire for information, strained relationships between family members and the mood of the patient all inform doctor–patient interactions and shape the physician’s decision-making in the building and the communicating of a diagnosis. Any distinction made between the ethical interest held within the act of communicating a diagnosis and the building of that diagnosis is thus an arbitrary one, as the following extract from a member of staff from a memory clinic describes:

It can be a discussion, “I don’t know what we’re going to find in this process but it may be this or there are these other causes.” You can ask, at this stage, “Are you the kind of person that likes to know or someone who would prefer not to know?” It makes it less start I think ‘cause you haven’t gone from nothing and then suddenly you’re saying the diagnosis. It can start to be floated or considered or thought about and unfold so by the time you’re bringing information together and this might be looking more like a dementia, say “Well look, I am starting to think we might be looking at this. We need to see how things go a bit.” It can evolve a bit more.

### What counts as ethical? Risk and mild cognitive impairment (MCI)

This restricted view of what counts as an ethical concern has historically been an interest of the social sciences (Haimes, 2002). In the quest to think differently about ethics and ethical engagement, sociology and the social sciences has attempted to question the taken-for-granted nature with which some aspects of bioethics are conceived. Empirical research and social science is able to raise ethical issues that would otherwise remain hidden. In the case of dementia diagnosis, the ethical impetus has been to achieve greater access to assessment and diagnosis for those with memory problems (Russell *et al,* 2013). Sociological empirical research is able to show the moral dilemmas produced as a result of an initial moral concern (see Price, 1997). In this case, the initial moral concern was one of equality and justice to ensure greater equity in the accessing of a dementia diagnosis. The consequence of this initial moral concern has been the increasing number of patients accessing assessment for problems with memory and cognition at an earlier stage. This has meant that there are increasing numbers of people being categorised as having a ‘pre-condition’ such as MCI, a condition that carries a significant degree of uncertainty. This uncertainty exacerbates the ethical issues to navigate between practitioners, patients and families.

The rights of patients to be fully informed, bound up in the principle of respect for autonomy, is particularly complex in situations where the information on offer carries so much uncertainty (Bharadwaj, 2002). Due to the poor predictive capacity of MCI for determining whether a person will progress on to develop dementia, it is difficult to determine what a diagnosis of MCI means. Is it part of getting older? Is it a diagnosis of a condition, or early dementia or is it a risk status indicating that you might get dementia in the future? (Bender, 2003). Such uncertainty is reflected in the accounts offered by clinicians who describe the difficulties they experience in making decisions regarding the sharing of information regarding risk:

I’m uneasy about that. With MCIs, there are no effective interventions…and then you’re leaving people with a diagnosis that they might get a horrible degenerative brain disease which we also can’t do anything about.

In this account, it is the practical and pragmatic concerns regarding risk, meaningful risk and its relationship with clinical intervention that creates the clinician’s feeling of unease regarding the communication of a pre-condition like MCI. This account reflects the grounded and pragmatic nature of ethical decision-making that occurs in the clinic. Such decision-making does not reside in a weighing up of conflicting ethical norms constituted as opposing binaries (Hoffmaster, 1992), but are instead based on the perceived meaning and usefulness of the information available to patients. The fuzziness of the boundaries separating MCI from dementia, and the implications that the different labels can have, means that concerns regarding the interests and circumstances of patients and families can become integral to the process of assigning such labels, as the following extract describes:

I’ve got a gentleman I’m going round to this afternoon now, he came to clinic, I think he was seen twice with mild cognitive impairment. Then he was re-referred and we saw him a couple of months ago, and again there was no change on his… well a very subtle change on the Addenbrooke’s. You know, he just dropped a couple of points but was still scoring really well. But just in my interaction with him, he was a bit sort of fluffy round the edges and you know when you get that feeling that this isn’t right for a very articulate, intelligent gentleman, and the wife was reporting, you know, that things were deteriorating a bit. But again, I think the doctor was a bit lacking in confidence to put a label on it and we’re still calling it mild cognitive impairment, but the wife is really unhappy with that and she rang them a couple of weeks ago with more evidence of how his functioning is going down. And so we have sort of said, well yes it could be early Alzheimer’s, so I’m taking the medication out ‘cause she was really keen to get him on the tablets.

Such a pragmatic approach also accounts for situational differences that occur in the decision-making practices of clinicians, where information may be perceived to have more or less benefit depending on a patient’s stage of life, their family circumstances or their perceived preparedness:

I did tell her she had MCI and didn’t know which way this would go, but, I felt, why distress her when there is no certainty, although I had a strong feeling (that it would progress). So it does depend on the preparedness, I am aware that I do make those judgements. I don’t give people information in the same way.

As the two previous examples illustrate, the communication of risk and the subsequent shaping of ethical practice has to be negotiated in processes of interaction between patients, doctors and families. In a similar vein to the diagnosis of a dementia, albeit with the uncertainty heightened, the navigation of moral dilemmas occurs through similar kinds of small, pragmatic strategies, so that the meaning attributed to a patient’s risk status is produced in collaborative processes, taking account of context and situational circumstances.

## Discussion

This paper challenges the assumption that there are distinct moments in which practitioners make ethical decisions, responding to and evaluating moral norms. The examples from the memory clinic interactions and the accounts of memory clinic staff highlight some key aspects of the diagnostic process and how these practices and interactions help in navigating moral concerns collaboratively, producing an ethics of diagnosis through the course of the clinical encounter. This article illustrates this through three key contributions. Firstly, the work pays attention to the socio-cultural and historical framing of moral norms, particularly in relation to autonomy and personhood, and shows how these are challenged by patient’s own accounts of the ways in which memory is experienced and by the meanings attached to memory and dementia. By doing this, the article highlights the tensions between the meanings of personhood produced through particular aspects of principalist ethics and the socially mediated nature of memory problems and their interpretation by individuals and families, illustrative of a personhood that is situated, embodied and relational. Such tensions and contradictions are shown to be present in the accounts of memory clinic staff, particularly when describing the ethics of their practice.

Secondly, the paper builds on existing literature to show the significance of the broader cultural and organisational contexts in which ethical concerns are negotiated, taking account of both disciplinary and organisational cultures that shape dementia diagnosis. This is important in shaping the ways in which diagnosis are reached which, when recognising the processual nature of ethical practice, has an important part to play in framing the communication of clinical information and the meanings attached to it. This attention to context and practices also highlights the small, pragmatic strategies through which practitioners respond to ethical problems and the processes through which clinicians, patients and families collaborate in the diagnostic process – shaping and moulding the ethicality of clinical consultations.

Finally, ethnography, as a method, is shown to provide distinct insights into the production of ethics in the clinic. Ethnography provides a means of getting inside everyday practices in order to achieve empathy and experience of what is being observed. Puig de la Bellacasa and Latimer (2013), in their paper on rethinking the ethical, shift the conception of ethics from the abstractions of traditional bioethics and instead forefront what they describe as moments of care whereby actors – in our case clinicians – attach themselves to particular ideas, accounts and materials. This does not renounce ethical engagement but rather locates ethics in practices thus adhering to an ethicality in process. The benefit of an ethnographic approach in this study is therefore in being able to identify practices that are experienced as showing care. For example, the pragmatic strategies for navigating moral concerns highlighted in the article suggest that extending moments of uncertainty and holding on to aspects of collectively in processes of assessment and diagnosis in the memory clinic may provide the resources for the production of ethical (or careful) practice (see also Kerr *et al,* 2007 for a similar argument regarding ambiguity in relation to the use of genetic research). Of course the benefits of an ethnographic approach, which enable this kind of care to be identified, are also indicative of its limitations, restricting the researcher’s ability to observe at a distance.

In summary, by providing an ethnographic account of ethical practice in the context of dementia diagnosis, this article builds on earlier work (Hoffmaster, 1992; Haimes, 2002; Hedgecoe, 2004) that makes a case for the contribution of medical sociology, ethnography and the social sciences more generally to the study of ethics in medical practice. This study highlights the practices that produce ethics in the context of dementia diagnosis, the social, clinical and organisational cultures that shape them and the collaborative and processual nature with which they are accomplished. Thus, to inform change and shape behaviour in the context of clinical practice necessitates a consideration of social relations, structures, processes and cultures. This ethnography of dementia diagnosis therefore illustrates how social scientists can contribute to an understanding of ethics, precisely by foregrounding contexts and practices.
